# Endoscopic neck surgery

**DOI:** 10.4103/0972-9941.30679

**Published:** 2007

**Authors:** P K Chowbey, Vandana Soni, R Khullar, Anil Sharma, M Baijal

**Affiliations:** Department of Minimal Access and Bariatric Surgery Centre, Sir Ganga Ram Hospital, New Delhi, India

**Keywords:** Endoscopic surgery, neck surgery, parathyroid, thyroid

## Abstract

Endoscopic surgery in the neck was attempted in 1996 for performing parathyroidectomy. A similar surgical technique was used for performing thyroidectomy the following year. Most commonly reported endoscopic neck surgery studies in literature have been on thyroid and parathyroid glands. The approaches are divided into two types i.e., the total endoscopic approach using CO_2_ insufflation and the video-assisted approach without CO_2_ insufflation. The latter approach has been reported more often. The surgical access (port placements) may vary-the common sites are the neck, anterior chest wall, axilla, and periareolar region. The limiting factors are the size of the gland and malignancy. Few reports are available on endoscopic resection for early thyroid malignancy and cervical lymph node dissection. Endoscopic neck surgery has primarily evolved due to its cosmetic benefits and it has proved to be safe and feasible in suitable patients with thyroid and parathyroid pathologies. Application of this technique for approaching other cervical organs such as the submandibular gland and carotid artery are still in the early experimental phase.

## INTRODUCTION

The cervical region comprises a plethora of well-defined anatomical structures systematically arranged in layers with minimal or negligible vascular overlap these well-defined layers form the vascular anatomical planes, which have been exploited by the endoscopic surgeon to create a working space for surgical manipulation. Reported initially in 1996,[1] endoscopic neck surgery has evolved in its application especially due to cosmetic benefits. The primary target organs have been the parathyroid and the thyroid glands,[2–9] although few studies have reported on its application to other cervical structures, such as the sub-mandibular gland and cervical spine.[9,10] Furthermore the approaches may be classified into total (pure) endoscopic (CO_2_ insufflation).[3–6] video- assisted endoscopic[11–14] and minimally invasive mini incision approaches.[15–18] The total endoscopic approach has been further sub-classified into a supraclavicular, anterior chest wall, axillary, and periareolar breast approach. The latter three have also been attempted in the video assisted endoscopic approach.

## ENDOSCOPIC PARATHYROIDECTOMY

Reported in 1996 by Gagner,[1] the parathyroid glands especially due to their size are amenable to the endoscopic approach. The drawback is their variable position. Minimally invasive parathyroidectomy has evolved due to a parallel progress in imaging and localizing techniques making a targeted approach possible.

The commonly performed localization studies are the 99^TC^ sestamibi scan and cervical ultrasound.[19–24] A combination of the sestamibi scan along with a radiological investigation has been described as equivalent to an open conventional bilateral exploration of the neck for localizing the parathyroid lesion. High-resolution cervical ultrasonography alone has reported a high success rate of 94% for pre-operative specific side localization of the parathyroid lesion.[20] The sensitivity was reported as 89% with a 98% positive predictive value.

The most popular minimally invasive approach for performing parathyroidectomy is the focused minimally invasive mini incision approach.[25,26] Few reports are available for total endoscopic parathyroidectomy, reporting on limited number of patients. Currently just below 50% of all parathyroidectomies are being performed by the minimal access approach popularly known as minimally invasive parathyroidectomy (MIP).[27] Restrictions in its applicability are selection criteria such as unilateral disease, (preferably a single adenoma), absence of thyromegaly, no previous neck surgery and no previous history of irradiation to the neck region.[28–30]

Techniques to ensure complete removal of the hyper functioning parathyroid tissue in MIP reported are intra-operative rapid parathormone assays,[29,31–34] frozen section and good clinical judgment followed by post-operative S.Ca^++^ and PTH level monitoring. Several studies have also reported day care MIP using local/regional anesthesia.[26] Such centers apply techniques, such as chemilumiscent assay for intact PTH level (quick PTH) giving a success rate of 95–98% to ensure a cure for the patient before discharge.[35–38] However, these results are best observed in patients with uniglandular disease. Provided a careful preoperative patient selection is performed, an MIP will cure the patient whether or not an intra operative QPTH assay is done [Table 1].[25]

**Table 1:** Complications reported in endoparathyroidectomy[39]

Recurrent laryngeal nerve palsy
Failed surgery (persistent - hyper calcemia/increase PTH)
Hemorrhage
Seroma
Hypocalcemia

Carbon dioxide embolization, a potential life threatening complication has so far not been reported.

Our own experience spans 8 years with 18 patients of primary hyperparathyroidism (PHPT) subjected to total endoscopic parathyroidectomy. About 17 of these patients were diagnosed with a single parathyroid adenoma on 99^TC^ sestamibi scan corroborated by an ultrasonography neck or an magnetic resonance imaging scan. One patient was diagnosed to have parathyroid hyperplasia. Ten procedures (7 procedures with CO_2_ insufflation and three procedures video-assisted) were performed by a supraclavicular approach, four by an anterior chest wall approach, and four by a periareolar breast approach. Carbon dioxide insufflation was maintained at 10 mm of Hg. Post-operative monitoring of S Ca^++^ and S PTH levels were done to confirm complete removal of all hyper functioning parathyroid tissue. There was one conversion due to non-localization of the parathyroid adenoma. The tumor was identified in the tracheo-esophageal groove. Although the number of patients in our experience is small, the results conform to those reported in literature in terms of safety and feasibility.

Our progress from a supraclavicular approach to a periareolar approach is strongly driven by superior cosmetic results, as the dissection involved in this approach is much more than a focused mini-incision approach.

## ENDOSCOPIC THYROIDECTOMY

Unlike parathyroidectomy, endoscopic thyroidectomy has progressed toward more remote sites of access to improve cosmesis and provide patients with a scar less neck. This has been more on patient demand as thyroid disease predominantly affects women. Endoscopic thyroidectomy was first reported in 1997[2] since then several reports have been published describing novel ways (neck, chest wall, axilla, and breast)[2–4,6,8,40,41] of access to this gland. Indications for endoscopic thyroidectomy in various studies include solitary, benign thyroid nodules, follicular and oxyphilic cell tumors, papillary micro carcinomas (< 1 cm in size and no evidence of clinical or radiological lymphadenopathy) and Grave's disease.[3,13,42] The latter has been reported only sporadically [Table 2].

**Table 2:** Exclusion criteria for endothyroidectomy[43–50]

Family history of Ca thyroid
History of neck irradiation
Significant thyroiditis
MNG
Thyroid nodule >35 mm (relative)

However, few reports describing the video assisted approach have reported removing tumor up to 74 mm in size.[12]

The aim of most studies apart from being cosmetically superior has been to be minimally invasive offering all associated advantages such as minimal post-operative pain, rapid recovery, and low analgesic requirement.[51–53]

In terms of invasiveness none of the distant sites of access prove to be truly minimally invasive as extent of invasion is much more compared to a focused, direct approach. The popularity has however persisted and increased due to improvement in cosmesis.

The supraclavicular approach has other advantages such as rapid access to thyroid (in the event of a vascular mishap), the advantage of applying external pressure for hemostasis.[54] The video-assisted focused approach using conventional instruments has a shorter learning curve.[55–58]

The size of the thyroid lobe removed has varied between 20–80 mm and the volume where recorded has ranged 15–73 grams in most studies.[16,42,58,59] A thyroid size beyond 70 mm or 70 grams becomes too voluminous to provide an adequate safe working space.

Studies reporting total endoscopic thyroidectomy with carbon dioxide insufflation have reported using ultrasonic shears for dissection and excision of the specimen.[5,6,40,58] The size and volume of tissue removed by either method is similar. The use of harmonic scalpel has been shown to reduce operative time in thyroid surgery [Table 3].[60]

**Table 3:** Post-operative complications with endothyroidectomy[55,59,61]

Rec. laryngeal n palsy
Hypocalcemia
Seroma
Hematoma
Wound infection
Subcutaneous emphysema (with CO_2_ insufflation)

All complications save the last have been reported with both video assisted and total endoscopic thyroidectomy, the latter results due to CO_2_ insufflation. Studies comparing intra operative pain and speed of recovery (return to normal activity) have all reported results in favor of the endoscopic approach[6,11,45,62] reaching statistical significance although analgesic requirement was not different.[52]

Both video-assisted and total endoscopic approaches have been reported for operating on thyroid cancer. The prerequisite are papillary tumors <1 cm in size with a negative clinical and radiological lymph node status.[11,12,45,55] Overall about 8% patients undergoing endoscopic thyroid surgery had papillary carcinoma. Patients of follicular carcinoma less than 5 cm may undergo endoscopic thyroidectomy. This surgery may also be recommended as a prophylaxis[63] to patients of multiple endocrine neoplasia with medullary carcinoma.

Our experience comprises 25 patients operated since 1997. About 21 patients had a solitary thyroid nodule and four patients had small multi-nodular goiter. In three patients a supraclavicular approach was adopted and 22 patients were operated by a peri-areolar approach. The surgery in one of the three patients of the supraclavicular approach was converted to a conventional exploration due to abnormally high vascularity of the gland, which turned out to be a multi-centric papillary carcinoma on histopathology. The patient subsequently underwent a completion thyroidectomy. Three patients developed subcutaneous emphysema which resolved over 24 hours and five patients showed bruising in the presternal region which resolved in 2 weeks. There were no other complications. It was easier operating from the periareolar approach as a larger working space was available. In 21 patients of solitary thyroid nodule a hemi thyroidectomy was performed and in three patients of multinodular goiter the excision extended to a little more than half the opposite lobe. The size of the resected specimen varied from 2 × 2.4 cm^2^ to 5 × 4.1 cm^2^ (the specimen were not weighed).

### The sub-mandibular gland and other structures

A few reports have been published over the past 2 years about an endoscopic approach to the sub-mandibular gland.[62,63] It has been demonstrated in cadaveric models to be possible.[64] Initial attempts, reported injury to facial artery and lingual nerve. Video-assisted approach deploying the harmonic scalpel has also been reported with a 15–20 mm skin incision. Endoscopic sentinel lymph node biopsy in oral malignancy is another area where this potential is being explored.[65] These reports are all in the very early phase and may at the most be described as experimental. The cervical spine is another region where endoscopy is being commonly practiced, but since it involves a specialty branch that is neuro surgery, it has not been discussed here.

## CONCLUSION

Endoscopic neck surgery offers a definite cosmetic advantage over its conventional counterpart. With increasing skill and patient demand, this surgery is going to be performed in more centers. However careful patient selection is advocated. Though few centers are reporting good results in thyroid malignancy, the role of endoscopy in thyroid malignancy is as yet controversial. Endoscopic approach to other neck structures such as the sub-mandibular gland is as yet in the experimental stage.

## References

[1] Gagner M (1996). Endoscopic subtotal para thyroidectomy in patients with primary hyperparathyroidism. Br J Surg.

[2] Yeung HC, Ng WT, Kong CK (1997). Endoscopic thyroid and parathyroidectomy surgery. Surg Endosc.

[3] Moural M, Pugin F, Elias B, Malaise J, Coche E, Jamar F (2002). Contribution of the video assisted approach to thyroid and parathyroid surgery. Acta Chir Belg.

[4] Ikeda Y, Takami H, Niimi M, Kan S, Sasaki Y, Takayama J (2002). Endoscopic thyroidectomy and parathyroidectomy by the axillary approach. A preliminary report. Surg Endosc.

[5] Maeda S, Shimizu K, Minami S, Hayashida N, Kuroki T, Furuichi A (2002). Video-assisted neck surgery for thyroid and parathyroid diseases. Biomed Pharmacother.

[6] Gagner M, Inabnet BW, Bierthoh L (2003). Endoscopic thyroidectomy for solitary nodules. Ann Chir.

[7] Ikeda Y, Takami H, Sasaki Y, Kan S, Niimi M (2000). Endoscopic neck surgery by the axillary approach. J Am Coll Surg.

[8] Cougard P, Osmak L, Esquis P, Ognois P (2005). Endoscopic thyroidectomy. A preliminary report including 40 patients. Ann Chir.

[9] Chantawibul S, Lokechareonlarp S, Pokawatana C (2003). Total video endoscopic thyroidectomy by an axillary approach. J Laparoendosc Adv Surg Tech A.

[10] Guyot L, Duroure F, Richard O, Lebeau J, Passagia JG, Raphael B (2005). Submandibular gland endoscopic resection: A cadaveric study. Int J Oral Maxillofac Surg.

[11] Ruggieri M, Straniero A, Pacini FM, Maiuolo A, Mascaro A, Genderini M (2003). Video assisted surgery of the thyroid diseases. Eur Rev Med Pharmacol Sci.

[12] Shimizu K, Tanaka S (2003). Asian perspective on endoscopic thyroidectomy: A review of 193 cases. Asian J Surg.

[13] Miccoli P, Berti P, Raffaelli M, Materazzi G, Baldacci S, Rossi G (2000). Comparison between minimally invasive video assisted thyroidectomy and conventional thyroidectomy: A prospective randomized study. Surgery.

[14] Gauger PG, Reeve TS, Delbridge LW (1999). Minimal access/minimally invasive parathyroidectomy. Br J Surg.

[15] Palazzo FF, Delbridge LW (2004). Minimal access/minimally invasive parathyroidectomy for primary hyperparathyroidism. Sirg Clin North Am.

[16] Gosnell JE, Sackett WR, Sidhu S, Sywak M, Reeve TS, Delbridge LW (2004). Minimal access thyroid surgery: Technique and report of the first 25 cases. ANZ J Surg.

[17] Malinvaud D, Potard G, Fortun C, Saraux A, Jezequel JA, Marianowski R (2004). Management of primary hyperthyroidism: Toward minimal access surgery. Joint Bone Spine.

[18] Lowney JK, Weber B, Johnson S, Doherty GM (2000). Minimal incision parathyroidectomy: Care, cosmesis and cost. World J Surg.

[19] Ben-Haim M, Zwas T, Mintz Y, Rosin D, Bar-Zakai B, Natur M (2003). Novel approach to parathyroid adenoma: Minimally invasive, focused, scan guided parathyroidectomy-experience from the first 100 cases. Harefuah.

[20] Gilat H, Cohen M, Feinmessr R, Benzion J, Shvero J, Segal K (2005). Minimally invasive procedure for resection of a parathyroid adenoma: The role of preoperative high resolution ultrasonography. J Clin Ultrasound.

[21] Yamashita H, Noguchi S (2005). Recent advances in the diagnosis and treatment of primary hyperparathyroidism. Nippon Geka Gakkai Zasshi [Japanese].

[22] Fuchs SP, Smits AB, de Hooge P, Muller AF, Gelissen JP, van Dalen T (2005). Minimally invasive parathyroidectomy: A good operative procedure for primary hyperparathyroidism even without the use of intraoperative parathyroid hormone assessment or a gamma probe. Ned Tijdschr Geneeskd.

[23] Lee JA, Inabnet WB (2005). The surgeon's armamentarium to the surgical treatment of primary hyperparathyroidism. J Surg Oncol.

[24] Rodriguez-Carranza S, Caceres M, Aguilar-Salinas CA, Gomez-Perez FT, Herrera MF, Pantoja JP (2004). Localization of parathyroid adenomasby (99 m) Tc-Sestamibir scanning: Upper neck versus lower neck lesions. Endocr Pract.

[25] Agarwal G, Barraclough B, Reene TS, Delbridge LW (2002). Minimally invasive parathyroidectomy using the “focused” lateral approach;2: Surgical technique. ANZ T Surg.

[26] Cohen MS, Finkelstein SE, Brunt LM, Haberfeld E, Kangrga I, Moley JF (2005). Outpatient minimally invasive parathyroidectomy using local / regional anaesthesia: A safe and effective operative approach for selected patients. Surgery.

[27] Sackett WR, Barraclough B, Reeve TS, Delbridge HW (2002). Worldwide trends in the surgical treatment of primary hyperparathyroidism in the era of minimally invasive parathyroidectomy. Arch Surg.

[28] Henry JK, Sebag F, Tamagnini P, Forman C, Silaghi H (2004). Endoscopic parathyroid surgery: Results of 365 consecutive procedures. World J Surg.

[29] Miccoli P, Bendinelli C, Vignali E, Mazzeo S, Cecchini GM, Pinchera A (1998). Endoscopic parathyroidectomy: Report of an initial experience surgery.

[30] Cougard P, Goudet P, Bilosi M, Peschaud F (2001). Videoendoscopic approach for parathyroid adenomas: Results of a prospective study of 100 patients. Ann Chir.

[31] Miccoli P, Berti P, Puccini M, Bendinelli C, Conte M, Picone A (1999). Video assisted parathyroidectomy: A series of 85 cases. Chirurgie.

[32] Henry JF, Sebag F, Maweja S, Hubbard J, Misso C, Da Costa V (2004). Video assisted parathyroidectomy in the management of patients with primary hyper parathyroidism. Ann Chir.

[33] Jortay AM, Verongstracte G, Wittersheim E, Hooghel L, Bisschop P, Bergmann P (2004). Intraoperative measurement of parathyroid hormone in minimally invasive surgery for parathyroid adenoma. Acta Otohinolaryngeal Belg.

[34] Schiffmann L, Mann B, Hotz H, Buhr HJ (2003). Minimal invasive surgery for pHPT - which patients will profit?. Zentrabl Chir.

[35] Irvin GL (1999). American Association of Endocrine Surgeons. Presidential address: Chasin' hormones.

[36] Irvin GL, Carneiro DM (2000). Management changes in primary hyperparathyroidism. JAMA.

[37] Carneiro DM, Irvin GL (2000). Late parathyroid function after successful parathyroidectomy guided by intraoperative hormone assay (QpTH) compared with the standard bilateral neck exploration. Surgery.

[38] Mekel M, Mahajna A, Ish-Shalom S, Barak M, Segal E, Salih AA (2005). Minimally invasive surgery for treatment of hyperparathyroidism. Isr Med Assoc J.

[39] Carty SE (2004). Prevention and management of complications in parathyroid surgery. Otolaryngol Clin N Am.

[40] Park YL, Han WK, Bae WG (2003). 100 cases of endoscopic thyroidectomy: Breast approach. Sur Laparosc Endosc Percu Tech.

[41] Takami H, Ikeda Y (2002). Minimally invasive thyroidectomy. ANZ Surg.

[42] Takami H, Ikeda Y (2003). Total endoscopic thyroidectomy. Asia J Surg.

[43] Takami HE, Ikeda Y (2006). Minimally invasive thyroidectomy. Curr Opin Oncol.

[44] Miccoli P, Berti P, Raffaelli M, Coute M, Materazzi G, Galleri D (2001). Minimally invasive video-assisted thyroidectomy. Am J Surg.

[45] Miccoli P, Minuto MN, Barellini L, Galleri D, Massi M, D'Agostino J (2004). Minimally invasive video assisted thyroidectomy - techniques and results over 4 years of experience (1992–2002). Ann Ital Chir.

[46] Miceoli P, Bellantone R, Mourad M, Walz M, Raffaelli M, Berti P (2002). Minimally invasive video-assisted thyroidectomy: Multiinstitutional experience. World J Surg.

[47] Bellantone R, Lombardi CP, Raffaelli MP, Boscherino M, de Crea C, Alesina PF (2002). Video assisted thyrodiectomy. Asia J Surg.

[48] Musella M, Lombardi S, Caiazzo P, Milone F, Di Palma R, de Franciscis S (2003). Video-assisted surgery of the thyroid: Outlines of the technique and analysis of the results. Ann Ital Chir.

[49] Palazzo FF, Sebag F, Henry JF (2006). Endocrine Surgical technique: Endoscopic thyroidectomy via the lateral approach. Surg Endosc.

[50] Gorness JE, Sackett WR, Sidhu S, Sywak M, Reeve TS, Delbridge LW (2004). Minimal access thyroid surgery: Technique and report of first 25 cases. ANZ J Surg.

[51] Mourad M, Pugin F, Elias B, Malaise J, Coche E (2002). Contributions of the video assisted approach to thyroid and parathyroid surgery. Acta Chir Belg.

[52] Gagner M, Inabnet WB (2001). Endoscopic thyroidectomy for solitary thyroid nodules. Thyroid.

[53] Bellantone R, Lombardi CP, Bossola M, Boscherim M, De Crea C, Alesina PF (2002). Video assisted vs conventional thyroid lobectomy: A randomized trial. Arch Surg.

[54] Inabnet WB, Gagner M, Gagner M, Inabnet WB (2002). Endoscopic thyroidectomy: Supraclavicular approach. Minimally Invasive Endocrine surgery.

[55] Bellantone R, Lombardi CP, Raffaelli M, Boscherini M, De Crea C, Traini E (2002). Video-assisted thyroidectomy. J Am Coll Surg.

[56] Kim JS, Kim KH, Ahn CH, Jeon HM, Kim EG, Jeon CS (2001). A clinical analysis of gasless endoscopoic thyrodiectomy. Surg Laparosc Endosc Percutan Tech.

[57] Shimizu K, Akira S, Jasmi AY, Kitamura Y, Kitagawa W, Akasu H (1999). Video-assisted neck surgery: Endoscopic resection of thyroid tumors with a very minimal neck wound. J Am Coll Surg.

[58] Yeh TS, Jan YY, Hsu BR, Chen KW, Chen MF (2000). Video-assisted endoscopic thyroidectomy. Am J Surg.

[59] Lombardi CP, Raffaelli M, Princi D, De Crea C, Bellantone R (2006). Videoassisted thyroidectomy: Report of a 7 year experience in Rome. Langenbecks Arch Surg.

[60] Mourad M, Saab N, Malaise J, Ngongang C, Fournier B, Daumerie C (2001). Minimally invasive video-assisted approach for partial and total thyroidectomy initial experience. Surg Endosc.

[61] Palazzo FF, Sywak MS, Sidhu SB, Delbridge LW (2005). Safety and feasibility of thyroid lobectomy via a lateral 2.5-cm incision with a cohort comparison of the first 50 cases: Evolution of a surgical approach. Langenbecks Arch Surg.

[62] Suzuki S, Takenoshita S (2006). Current topics endoscopic surgery for thyroid cancer. Nippon Geka Gakkai Zassli.

[63] Komatsuzaki Y, Ochi K, Sugiura N, Hyodo M, Okamoto A (2003). Video-assisted submandibular sialadenectomy using an ultrasonic scalpel. Auris Nasus Larynx.

[64] Kessler P, Bloch-Birkholz A, Birkholz T, Neukam FW (2006). Feasibility of an endoscopic approach to submandibular neck region – experimental and clinical results. Br J Oral Maxillofac Surg.

[65] Guyot L, Duroure F, Richard O, Lebeau J, Passagia JG, Raphael B (2005). Submandibular gland endsocopic resection: A cadaveric study. Int J Oral Maxillofax Surg.

[66] Pedachenko EG, Tanaseichuk AF, Khizhniak MV, Pedachenko IuE (2003). Endoscopic microsurgery in cervical disc hernias. Zh Vopr Neirokhir Im N N Burdenko [Russian].

[67] Sipperstein AE, Berber E, Morkoyun E (2002). The ise o the harmonic scalpel vs conventional knot lying for vessel ligation in thyroid surgery. Arch Surg.

[68] Pitman KT, Sisk JD (2006). Endoscopic sentinel lymph node biopsy in a porcine model. Laryngoscope.

